# Confinement Effects on Polymer Dynamics: Thermo-Responsive Behaviours of Hydroxypropyl Cellulose Polymers in Phospholipid-Coated Droplets (Water-in-Oil Emulsion)

**DOI:** 10.3390/polym9120680

**Published:** 2017-12-06

**Authors:** Kazunari Yoshida, Keitaro Horii, Azusa Saito, Akito Takashima, Izumi Nishio

**Affiliations:** 1 Department of Mechanical Systems Engineering, Graduate School of Science and Engineering, Yamagata University, 4-3-16, Jonan, Yonezawa 992-8510, Yamagata, Japan; azusasaito@yz.yamagata-u.ac.jp; 2 Department of Physics and Mathematics, College of Science and Engineering, Aoyama Gakuin University, 5-10-1 Fuchinobe, Chuo-ku, Sagamihara 252-5258, Kanagawa, Japan; horii.k13@nishio-lab.net (K.H.); takashima.a.aa@phys.aoyama.ac.jp (A.T.); izumi24058@yahoo.co.jp (I.N.)

**Keywords:** dynamics, thermo-responsive polymer, water-in-oil droplet (emulsion), artificial cell model

## Abstract

In order to construct the artificial cells and to understand the physicochemical properties of living cells, it is important to clarify the cell-sized confinement effect on the behaviours of bio-inspired polymers. We report the dynamic behaviours of aqueous hydroxypropyl cellulose (HPC) solution coated with phospholipids in oil (water-in-oil droplets, W/O droplets), accompanied by an increase in the temperature. We directly observed the beginning of phase separation of HPC solution using a fluorescence microscope and confirmed the dependence of such phenomena on droplet size. The results indicate that the start time of phase separation is decreased with an increase in droplet size. The experimental results suggest that the confinement situation accelerates the phase separation of aqueous HPC solutions.

## 1. Introduction

Recently, a physicochemical approach to artificial cells has been studied with a view to its applications in medical science, in addition to the research of embryonic stem (ES) [1,2] and induced pluripotent stem (iPS) [3] cells. It is beneficial to utilize the preparation methods of the liposome [4,5,6] and the water-in-oil (W/O) droplet coated by phospholipid [7,8] for constructing an artificial cell because such systems are representative simple model systems of living cell and/or cell membranes and can be prepared easily. It is known that living cells possess the various types of polymers with high concentration, and biochemical reaction and folding of protein might be influenced by crowding [9]. Hence, it is very important to clarify the physicochemical effect of confinement on behaviours of polymers.

In the 1970s, it was revealed that coil-globule transition of a single polymer chain was induced by various stimuli such as change of solvent composition [10]. Helix-coil transitions of biopolymers has been also studied for many years [11,12,13]. Phase separation of polymers has been reported, in addition to the phase transition [14,15]. It is speculated that such fundamental properties and behaviours of polymers are also influenced by crowding.

There are many studies for clarifying the effect of confinement on behaviours of polymers to date. For instance, the group of Dimova and Lipowsky has revealed the contact angle between polymer-rich and water-rich phases enclosed by lipid bilayer vesicles [16,17,18,19]. Several resemble studies are also reported for clarifying the interfacial phenomena in confined phase-separated polymer solution [20,21]. Kato et al. reported that gene expression is significantly influenced by confinement, utilizing W/O droplet coated with phospholipids [22]. Further studies of confinement effect on dynamic behaviours of polymers is required for establishing artificial cells.

Hydroxypropyl cellulose (HPC) is one of the representative biocompatible polymers since its main component is a multi-sugar chain, and the living cell possesses various kinds of sugar derivative molecules such as glycolipids [23]. The phase behaviours of aqueous HPC solution have been widely studied utilizing spectroscopic methods [24,25,26]. The results of previous studies show that HPC solutions exhibit a lower critical solution temperature (LCST) at ca. 42 ${}^{\circ }$C [25,26]. Furthermore, it has been shown that volume phase transition of HPC hydrogels is also significantly correlated with the LCST [27,28,29]. However, dynamic phase behaviour of HPC solution enclosed by phospholipids is still ambiguous.

In this study, we report the relationship between droplet size and dynamic behaviour of aqueous HPC solution. The results of the experiments indicate that the starting time of phase separation of HPC solution is significantly correlated with droplet size.

## 2. Materials and Methods

### 2.1. Materials

Mineral oil was obtained from Nacalai Tesque, Inc. (Kyoto, Japan). Such mineral oil has been widely used in the studies of W/O droplets [8,21]. Ultra-pure water was obtained using a WT101UV Autopure system of Yamato Scientific Co., Ltd. (Tokyo, Japan). Electrical resistivity of the obtained ultra-pure water was more than 18 MΩ·cm at 25 ${}^{\circ }$C. HPC (2.0–2.9 mPa·s), calcein (fluorescent dye, $\lambda_{\mathrm{ex}}/\lambda_{\mathrm{em}}=495\;\mathrm{nm}/515\;\mathrm{nm}$) and chloroform were obtained from Wako Pure Chemical Industries, Ltd. (Osaka, Japan). 1,2-dipalmitoyl-sn-glycero-3-phosphocholine (DPPC) was obtained from Avanti Polar Lipids, Inc. (Birmingham, AL, USA). Rhodamine B 1,2-dihexadecanoyl-sn-glycero-3-phosphoethanolamine, triethylammonium salt (rhodamine DHPE, fluorescent lipid, $\lambda_{\mathrm{ex}}/\lambda_{\mathrm{em}}=560\;\mathrm{nm}/580\;\mathrm{nm}$), was obtained from Invitrogen (Carlsbad, CA, USA).

### 2.2. Preparation of HPC Solution Droplets

First, DPPC (2 mM) and small amount of rhodamine DHPE were dissolved in chloroform (105 μL), and the lipid solution was poured into a small test tube. Then, the solvent of the solution was removed to form a thin dry lipid film under vacuum conditions. A volume of 400 μL of mineral oil was poured into the test tube with lipid film, and then, the tube was sonicated for 90 min at 60 ${}^{\circ }$C to disperse the lipids all over the oil. Finally, 40 wt % of aqueous HPC solution (40 μL) was added to the lipid and oil solution, and emulsification was performed via sonication for 10 min and pipetting. The final concentration of rhodamine DHPE and calcein were 0.01 mM (calculated) in the mineral oil and 0.1 mM (measured) in the aqueous HPC solution, respectively. The HPC solution droplets are stable in several days, and the droplets were observed within a day from that those ware made. The droplet sized are up to 130 μm in diameters, and the deviation of size is shown below (Results and Discussion).

### 2.3. Microscopic Observation under Temperature Control

**Figure 1:**
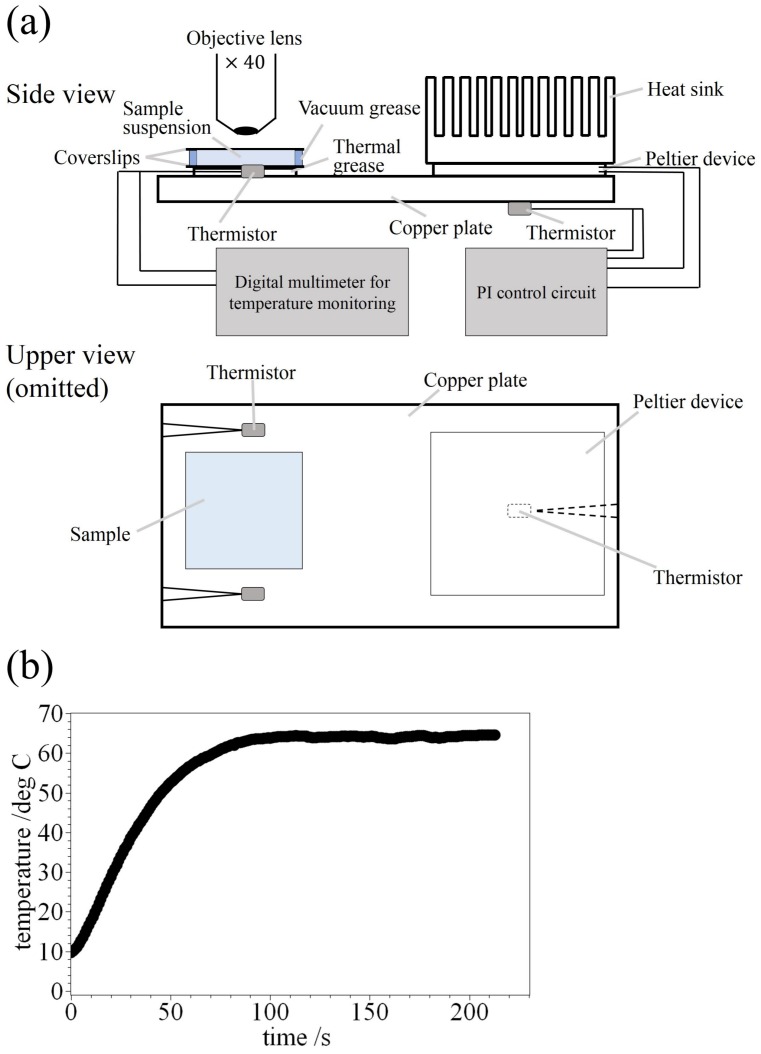
(**a**) Schematic illustration of temperature-control system. We used three thermistors; the two were for temperature monitoring, and the other was for feedback in order to control the plate temperature. The temperature of the copper plate was controlled through a proportional integral (PI) circuit and a Peltier device (deviation < 0.1 ${}^{\circ }$C). The measured temperature was defined as an averaged value using two thermistors. Thickness of the sample suspension is within 1 mm; (**b**) The thermal profile was measured by thermistors in the case of the situation from 10 ${}^{\circ }C$ to 65 ${}^{\circ }C$. The temperature reached above ca. 42 ${}^{\circ }C$ within 50 s from the beginning of heating.

A volume of 20 μL of sample suspension including HPC solution droplets was put between two glass coverslips and sealed with vacuum grease. The sample cell was put on the hand-made temperature-control system (deviation < 0.1 ${}^{\circ }$C, see Figure 1a). We used three thermistors; the two were used for temperature monitoring, and the other was for feedback in order to control the plate temperature. The temperature of copper plate was controlled through a proportional integral (PI) circuit and a Peltier device (deviation < 0.1 ${}^{\circ }$C). The measured temperature was defined as an averaged value using two thermistors. The thickness of the sample suspension is less than 1 mm. The temperature control system is shown in Figure 1a. We observed the HPC solution droplets on the temperature-control system using a fluorescence microscope, BX40 (Olympus, Tokyo, Japan) equipped with a ×40 objective lens (Olympus, Japan), a digital camera DP73 (Olympus, Japan) and a Hg lamp. We observed the lipid monolayer (rhodamine DHPE) using a Olympus WIG filter set ($\lambda_{\mathrm{ex}}/\lambda_{\mathrm{em}}=$ 520–550 nm/above 580 nm) and the HPC solution (inside the droplets, calcein) using a Olympus NIBA filter set ($\lambda_{\mathrm{ex}}/\lambda_{\mathrm{em}}=$ 470–490 nm/510–550 nm). The dynamics of the HPC solution droplet was observed when we changed the temperature from 10 ${}^{\circ }$C to 65 ${}^{\circ }$C (see Figure 1b). The videos were recorded at 7 fps.

## 3. Results and Discussion

**Figure 2:**
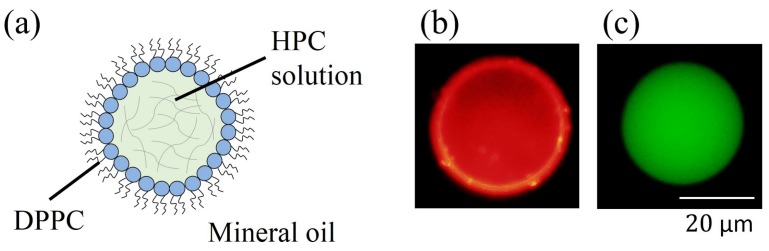
(**a**) Schematic illustration of HPC solution droplet (W/O type dorplet); (**b**) Microscopic images in the case of observation of dyes rhodamine DHPE, $\lambda_{\mathrm{em}}=580\;\mathrm{nm}$ (red), corresponding to place of lipids; (**c**) Microscopic images in the case of observation of dyes calcein, $\lambda_{\mathrm{em}}=515\;\mathrm{nm}$ (green), corresponding to place of water molecules inside the droplet, and in this photograph, the water molecules (also the HPC) are uniformly dispersed.

Figure 2a shows the schematic illustration of HPC solution droplet which is water-in-oil (W/O) type droplet. Such droplets can be formed through the procedure as shown in previous section. We confirmed the such mesoscopic structure using a optical microscopy (see Figure 2b,c). Figure 2b indicates that rhodamine DHPE is mainly localized in the rim of the droplets while calcein is mainly localized inside of the droplets. These facts suggest that the HPC droplets floating in the oil phase are coated with DPPC lipids since rhodamine DHPE and calcein are mainly localized in phospholipid-rich [7] and water-rich [30] regions, respectively.

**Figure 3:**
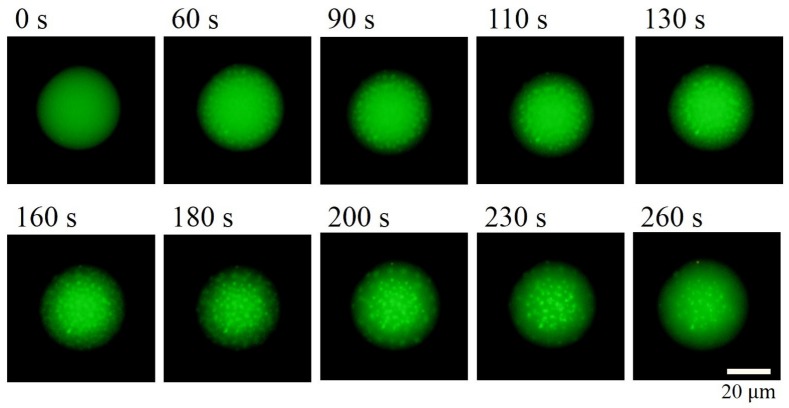
Typical microscopic images of hydroxypropyl cellulose (HPC) droplet with elapsed time when we changed the temperature from 10 ${}^{\circ }C$ to 65 ${}^{\circ }C$. The dot-like phase-separation pattern appeared with an increase in temperature. The phase separation progressed from the interface to the centre of the droplet. The bright region corresponds to the water-rich phase, while the dark region inside the droplet corresponds to the HPC-rich phase.

Figure 3 shows typical time-lapse images of HPC droplet, observed by fluorescence microscopy when we changed the temperature from 10 ${}^{\circ }C$ to 65 ${}^{\circ }C$. With increasing elapsed time, the dot-like pattern appeared inside the droplet. This indicates that phase separation was caused by increase in the temperature since the HPC has LCST in the aqueous solution [25,26]. The bright region corresponds to water-rich phase while the dark region (inside droplet) corresponds to HPC-rich phase since calcein can be dissolved in aqueous solution [30]. This result shows that the phase separation progressed from the interface to the centre of the droplet. This phenomenon suggests that the head groups of lipids influenced the phase dynamics of HPC solution.

**Figure 4:**
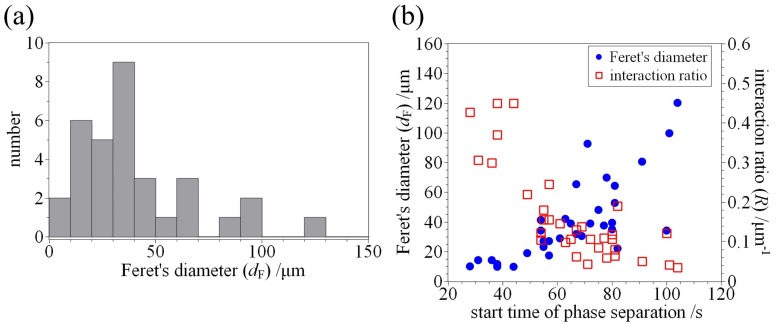
(**a**) Histogram plot of Feret’s diameter ($d_{F}$) of HPC droplets (droplet size, n=33). The value of Feret’s diameter is 34±27μm (median ± SD); (**b**) Relationship between start time of phase separation and $d_{F}$ of HPC droplets/interaction ratio between HPC and 1,2-dipalmitoyl-sn- glycero-3-phosphocholine (DPPC) (*R*).

We investigated the Feret’s diameters ($d_{F}$), which are the maximum caliper of HPC droplet (droplet size), derived from cross section images using a software ImageJ [31]. Figure 4a shows the histogram of the Feret’s diameters (n=33). The result of statistical analysis shows that HPC droplet size is 34±27μm (median ± SD). We are able to prepare the HPC droplets with up to 130μm using this emulsification procedure.

Next, we investigated the size dependency of the start time of phase separation on droplet sizes/interaction ratio between HPC and DPPC (*R*). As shown above, phase separation is induced by an increase in temperature, and the elapsed time when the temperature begins to be increased from 10 ${}^{\circ }C$. This corresponds to easiness of phase separation (velocity of phase separation dynamics). Furthermore, we also defined the interaction ratio between HPC and DPPC as follows:

(1) $$R=\frac{\pi d_{F}}{\pi d_{F}^{2}/4\pi }=\frac{4\pi }{d_{F}},$$

which is the perimeter to area of cross section ratio of HPC droplets. Figure 4b shows the relationship between start time of phase separation and droplet size/interaction ratio between HPC and DPPC. The start time of the phase separation is increased with an increase in droplet size. This result indicates that a longer time is required for the phase separation in the case of larger HPC droplets. In contrast, the interaction ratio is decreased with an increase in start time of phase separation, which indicates that a shorter time is required for the phase separation in the case of higher interaction between HPC and lipids. These facts suggest that the confinement situation accelerates the phase separation of aqueous HPC solutions although the effect of size dependency of thermal conductivity is ignorable. For the relationship between interaction ratio *R* and start time of phase separation (see Figure 4), it is considered that the lipid head groups influenced the dynamic phase behaviours of aqueous HPC solution. In other words, there is a possibility that DPPC lipids coating the HPC droplets have an ability to accelerate the phase separation. This is in good agreement with the fact that the phase separation progressed from the interface of the droplet (see Figure 3). To confirm such a possibility, further experiments are required. However, to the best of our knowledge, this is the first report showing the effect of droplet size/lipids on dynamic phase behaviour of aqueous HPC solutions.

For the present results of relationship between *R* and start time of phase separation, it is considered that the lipid head groups influence the movement of HPC polymers and water molecules. In this study, the DPPC, which has a zwitterion in the head group, has been used for coating the droplet. The HPC polymers hardly bind to the lipids coated interface since the zwitterion, including bio-inspired materials, have been widely investigated for anti-fouling materials [32]. Hence, further experiments using lipids possessing other types of head groups, such as 1,2-dipalmitoyl-sn-glycero-3-phospho-(1${}^{\prime }$-rac-glycerol) (DPPG), are required in order to clarify the effects of interface on the interactions between HPC, lipids and water molecules.

Previously, Kato et al. reported that gene expression rate (protein production rate) is proportional to 1/r, where *r* is the radius of the confinement droplet radius. This means that protein expression is accelerated in a smaller confinement space [22]. We consider that the acceleration of this biochemical gene expression and phase separation of the polymer is very important information to construct the artificial cells and to understand the living cells. Hence, such investigations may lead to further understanding of the optimum size of an artificial cell for constructing the various types of cells.

Kurokawa et al. reported that the artificial cytoskeleton made with deoxyribonucleic acid (DNA) stabilizes the artificial cells [33]. In addition, Terasawa et al. reported that the budding of lipid bilayer vesicles (similar to cell division) is induced by depletion effect by polymers inside the vesicles [34]. We have to combine the knowledges obtained from previous studies and the present study in order to construct the artificial cells and to understand the physicochemical properties of living cells.

## 4. Conclusions

In summary, we investigated the dynamic phase behaviours of aqueous hydroxypropyl cellulose (HPC) solution coated with phospholipids in oil (water-in-oil droplets, W/O droplets), accompanied with an increase in the temperature. The experimental results indicate that the start time of phase separation of HPC droplets is strongly correlated with droplet size/interaction ratio between HPC and DPPC. It is considered that the confinement situation accelerates the phase separation of aqueous HPC solutions. In addition, the importance of lipid head group coating droplets was also suggested. Hence, in order to understand the detailed effect of lipids on polymer dynamics, further experiments are required using different types of lipids. The finding of this study and the results of further experiments may lead to a detailed understanding of the optimum size of artificial cells and then to a new range of studies of artificial cell and detailed understanding of the physicochemical properties of living cells.

## Abbreviations

The following abbreviations are used in this manuscript:

HPC	hydroxypropyl cellulose
W/O droplets	water-in-oil droplets
DPPC	1,2-dipalmitoyl-sn-glycero-3-phosphocholine
DPPG	1,2-dipalmitoyl-sn-glycero-3-phospho-(1${}^{\prime }$-rac-glycerol)
